# Red Cell Distribution Width and Other Red Blood Cell Parameters in Patients with Cancer: Association with Risk of Venous Thromboembolism and Mortality

**DOI:** 10.1371/journal.pone.0111440

**Published:** 2014-10-27

**Authors:** Julia Riedl, Florian Posch, Oliver Königsbrügge, Felix Lötsch, Eva-Maria Reitter, Ernst Eigenbauer, Christine Marosi, Ilse Schwarzinger, Christoph Zielinski, Ingrid Pabinger, Cihan Ay

**Affiliations:** 1 Clinical Division of Haematology and Haemostaseology, Department of Medicine I, Comprehensive Cancer Center Vienna, Medical University of Vienna, Vienna, Austria; 2 Center for Medical Statistics, Informatics, and Intelligent Systems, Medical University of Vienna, Vienna, Austria; 3 Clinical Division of Oncology, Department of Medicine I, Comprehensive Cancer Center Vienna, Medical University of Vienna, Vienna, Austria; 4 Department of Laboratory Medicine, Medical University of Vienna, Vienna, Austria; Maastricht University Medical Center, Netherlands

## Abstract

**Background:**

Cancer patients are at high risk of developing venous thromboembolism (VTE). Red cell distribution width (RDW) has been reported to be associated with arterial and venous thrombosis and mortality in several diseases. Here, we analyzed the association between RDW and other red blood cell (RBC) parameters with risk of VTE and mortality in patients with cancer.

**Methods:**

RBC parameters were measured in 1840 patients with cancers of the brain, breast, lung, stomach, colon, pancreas, prostate, kidney; lymphoma, multiple myeloma and other tumor sites, that were included in the Vienna Cancer and Thrombosis Study (CATS), which is an ongoing prospective, observational cohort study of patients with newly diagnosed or progressive cancer after remission. Primary study outcome is occurrence of symptomatic VTE and secondary outcome is death during a maximum follow-up of 2 years.

**Results:**

During a median follow-up of 706 days, 131 (7.1%) patients developed VTE and 702 (38.2%) died. High RDW (>16%) was not associated with a higher risk of VTE in the total study cohort; in competing risk analysis accounting for death as competing variable the univariable subhazard ratio (SHR) was 1.34 (95% confidence interval [CI]: 0.80–2.23, p = 0.269). There was also no significant association between other RBC parameters and risk of VTE. High RDW was associated with an increased risk of mortality in the total study population (hazard ratio [HR, 95% CI]: 1.72 [1.39–2.12], p<0.001), and this association prevailed after adjustment for age, sex, hemoglobin, leukocyte and platelet count (HR [95% CI]: 1.34 [1.06–1.70], p = 0.016).

**Conclusions:**

RDW and other RBC parameters were not independently associated with risk of VTE in patients with cancer and might therefore not be of added value for estimating risk of VTE in patients with cancer. We could confirm that high RDW is an independent predictor of poor overall survival in cancer.

## Introduction

Red cell distribution width (RDW) is a parameter of the complete blood count (CBC) that describes the size variation of red blood cells (RBC). It is routinely measured by most of the modern hemocytometers and is calculated by dividing the standard deviation of the mean corpuscular volume (MCV) by the respective actual MCV, and is expressed as percentage. A high RDW represents a large variation of the RBC volume, called anisocytosis, and is found in conditions with an increased number of small or large RBC. Hence, RDW can be used to discriminate between different forms of anemia, since iron deficiency anemia or megaloblastic anemia are accompanied with elevated RDW, whereas in thalassemia RDW is usually normal [1]–[3]. Other parameters routinely given by CBC that provide information about RBC are hematocrit, hemoglobin concentration, MCV, mean corpuscular hemoglobin (MCH) and mean corpuscular hemoglobin concentration (MCHC).

Besides the conventional use of RDW for discriminating between different forms of anemia, a number of studies have suggested that RDW could be a potentially useful marker in a variety of other diseases, such as heart failure [4], atrial fibrillation [5], lung cancer [6] or inflammatory disorders [7], frequently associated with a worse prognosis. Also an association between high RDW and risk of cardiovascular thrombotic disorders, as well as with increased mortality in patients with cardiovascular diseases has been described [1]. Interestingly, in a population-based study high RDW was reported to predispose to venous thromboembolism (VTE) [8]. Two case-control studies found a high RDW in patients with deep venous thrombosis [9], [10]. Furthermore, high RDW was correlated with a poor outcome in patients who suffered pulmonary embolism [11]. Moreover, in a meta-analysis of seven community-based studies of older individuals high RDW was described to increase risk of mortality [12]. In a subgroup analysis of the latter study RDW was also associated with an increased risk of death in patients with cancer.

Studies that have investigated other RBC parameters and their association with the risk of thrombosis are limited. In non-cancer patients, a high hematocrit was found to be associated with increased risk of first [13] and recurrent VTE [14]. Increasing levels of MCV and MCH were associated with VTE in a case-control study [9]. In patients with cancer, a low hemoglobin level was reported to be a risk factor for development of VTE [15].

As patients with cancer have a high risk of developing VTE, a complication that is associated with high morbidity and mortality [16], a number of studies over the past years have focused on the identification of laboratory and clinical parameters associated with risk of VTE. Khorana et al. developed a scoring model for risk stratification of VTE in patients with cancer [15] that includes laboratory parameters of CBC (hemoglobin levels, leukocyte count, platelet count). This risk scoring model could be validated in subsequent studies [17], [18].

While previous studies indicated that RDW or hematocrit might have a predictive value for risk of VTE in the general population [8], [13] no data are available for patients with cancer. Therefore, we aimed to investigate whether RDW, hematocrit and other RBC parameters are associated with the development of VTE in patients with cancer. Furthermore, we analyzed the association between RBC parameters and mortality in patients with different types of cancer included in our study.

## Materials and Methods

### Study design and study population

This study was performed within the framework of the Vienna Cancer and Thrombosis Study (CATS), an ongoing prospective, single-centre, observational cohort study that started in 2003 at the Medical University of Vienna, Austria. Patients with newly diagnosed cancer or progression of disease after remission are included in the study and followed for a maximum of 2 years. Exact methods, inclusion and exclusion criteria of CATS have been described in previous publications [19], [20]. Briefly, patients with cancer of the brain, breast, lung, upper or lower gastrointestinal tract, pancreas, kidney, prostate or gynecological system; sarcoma and hematologic malignancies (lymphoma, multiple myeloma) were included in the study. Patients with a thromboembolic event or chemotherapy within the last three months, surgery or radiotherapy within the last two weeks or continuous anticoagulation with vitamin K antagonists or low molecular weight heparin were excluded. After written informed consent, a structured interview about the patients' medical history was performed and a single blood sample was drawn. Follow-up was performed regularly approximately every 3 months as described in previous publications [19], [20]. The observation period started from the time of blood sampling and lasted for 2 years or until the occurrence of VTE, death, loss of follow-up or withdrawal of consent. Once a year, the Austrian death registry was searched for entries about study participants.

No routine screening for VTE was performed. Diagnosis of VTE had to be confirmed by duplex sonography or venography for deep vein thrombosis (DVT) or by computed tomography or ventilation/perfusion lung scan for pulmonary embolism (PE), respectively. In patients who died during follow-up, death certificates and, if available, autopsy findings were checked for diagnosis of fatal PE.

Until December 2013, 2030 patients had been included in the study. Of this cohort, 190 patients had to be excluded, either because they did not fulfill the exact in- and exclusion criteria after re-evaluation (n = 94), or because no follow-up information (n = 87) or no adequate materials for laboratory measurements were available (n = 9).

#### Healthy control subjects

Healthy individuals (n = 25, median age: 56 years [25^th^–75^th^ percentile: 46–60], 17 [68%] female) without a history of venous or arterial thrombosis and cancer from the same geographic region served as controls for comparison of RBC/RDW parameters.

### Ethics statement

Ethical approval for the study was received from the institutional ethics committee of the Medical University of Vienna and the study has been conducted in accordance with the Declaration of Helsinki.

### Outcome measure of the study

Primary endpoint of the study was occurrence of symptomatic or fatal VTE. The secondary outcome measure was death of any cause within 2 years after study inclusion.

### Blood sampling and laboratory analysis of RDW

At the day of study inclusion, venous blood samples were collected in Vacutainer K3-EDTA tubes (Vacuette; Greiner-Bio One) by sterile venipuncture and analyzed within 2 hours from sampling. RBC parameters were routinely determined using a Sysmex XE-2100 hematology analyzer. RDW-CV (coefficient of variance) was used for all analyses in the current study. RDW-CV was calculated automatically by dividing the standard deviation of the MCV through the actual MCV, multiplied by 100. This allows illustration of size variation of red blood cell size around their mean size (i.e. around the MCV), expressed as percentage. The reference range for RDW-CV in the routine lab of the Vienna General Hospital of the Medical University of Vienna was based on evaluation of this parameter in the local normal, healthy population according to standardized procedures. The reference range of RDW at our hospital is 11%–16%.

### Statistical analysis

Statistical analyses were performed using SPSS (Windows Version 21.0, Armonk, NY: IBM Corp.), STATA (Windows Version 12.0, Stata Corp., TX, USA), and R (Windows Version 3.0.2., R Core Development Team). Continuous variables were summarized with the median and the interquartile range (IQR), and categorical variables by absolute frequencies and percentages. The correlation between continuous variables was assessed using Spearman's correlation coefficient. The Kruskal-Wallis test was used to test for differences in continuous variables between the different tumor types. The Wilcoxon-Mann-Whitney test was used to test for differences in continuous variables between patients and healthy controls and between patients with different cancer stages.

Median survival time was calculated using the reverse Kaplan-Meier method. Cumulative incidence functions (CIFs) for VTE risk with point estimates and 95% confidence intervals were estimated non-parametrically using the estimators proposed by Marubini & Valsecchi, and Choudhury, respectively (implemented in Stata's stcompet suite). Death-from-any-cause was incorporated as the competing event of interest in all following CR calculations. CIFs between patients with high and low baseline RDW were compared using Gray's test (CumIncidence package in R). Univariable and multivariable proportional subdistribution hazards regression models for competing risk data according to Fine and Gray [21] were used to model the risk of VTE. The multivariable competing risk regression analyses comprised the variable of main interest (RDW or other RBC parameters), either analyzed as a continuous variable or divided into four groups according to quartiles of the variable in the corresponding study population, and it comprised also the following variables: In the first model we adjusted for age and sex, in the second model for age, sex and different groups of tumors (divided into localized solid tumors, solid tumors with distant metastasis, brain tumors and hematological malignancy); in the third model we adjusted for age, sex and use of erythropoiesis-stimulating agents (ESA) during the time period of one month before until 3 months after entry into the study; and the fourth model was adjusted for age, sex, hemoglobin level, leukocyte count and platelet count. Continuous co-variables were centered at the mean (age, hemoglobin). To allow illustration with cumulative incidence curves, we defined 2 groups of patients with high or non-elevated RDW. The cut-off for this categorized variable was set at 16%, which is the upper limit of the reference range for RDW (reference range: 11%-16%) in our hospital's routine laboratory.

Univariable and multivariable Cox Regression models were used to evaluate the influence of RDW and other RBC parameters on overall survival. Death of any cause was considered as the event of interest. Data were censored at the end of the study period (after two years) or loss to follow-up. The multivariable Cox regression models comprised the same variables as the above-described competing risk regression analyses. For visualization of survival probabilities in patients with high or non-elevated RDW, Kaplan-Meier curves were used, again taking RDW>16% as the cut-off for categorization.

Two-sided p*-*values less than 0.05 were regarded as statistically significant. No adjustment for multiplicity was done. The proportional (sub-)hazards assumption was examined for both VTE and mortality models by fitting an interaction between each variable of interest and the natural logarithm of continuous follow-up time. No evidence for interactions between the study variables was observed.

## Results

### Study Population

Table 1 shows the detailed characteristics of the patients included in the study (n = 1840; 843 [45.8%] female and 997 male [54.2%]). The median age of the total study population was 62 years (25^th^–75^th^ percentile: 52–68). A solid tumor was diagnosed in 1286 (69.9%) patients, 245 patients (13.3%) had a brain tumor (mainly high grade glioma) and 309 (16.8%) had a hematological malignancy (lymphoma or multiple myeloma). Twenty-five healthy individuals (median age: 56 years [25^th^–75^th^ percentile: 46–60], 17 [68%] female) from the same geographical region served as controls.

**Table 1:** Characteristics of the total study population, of patients who developed venous thromboembolism (VTE) and of patients who died during the observation period, as recorded at the time of entry into the study.

		All study patients n = 1840	VTE during follow-up^a^ n = 131 (7.1%)	Death during follow-up^a^ n = 702 (38.2%)
Median Age, y (IQR)		62 (52–68)	61 (51–67)	63 (55–70)
Sex, n				
	Female	843	55 (6.5%)	284 (33.7%)
	Male	997	76 (7.6%)	418 (41.9%)
Site of cancer, n				
	Lung	309	21 (6.8%)	194 (62.8%)
	Breast	273	7 (2.6%)	45 (16.5%)
	Lymphoma	260	12 (4.6%)	40 (15.4%)
	Brain	245	32 (13.1%)	131 (53.5%)
	Colon/Rectum	182	14 (7.7%)	63 (34.6%)
	Prostate	157	3 (1.9%)	31 (19.7%)
	Pancreas	130	19 (14.6%)	84 (64.6%)
	Stomach	63	8 (12.7%)	45 (71.4%)
	Kidney	42	1 (2.4%)	10 (23.8%)
	Multiple myeloma	49	3 (6.1%)	6 (12.2%)
	Others	130	11 (8.5%)	53 (40.8%)
Newly diagnosed vs. recurrent disease, n				
	Newly diagnosed	1353	89 (8%)	486 (35.9%)
	Recurrent disease	487	25 (5.9%)	216 (44.4%)
Tumor group, n				
	Localized solid tumor	715	40 (5.6%)	153 (21.4%)
	Solid tumors with distant metastasis	571	44 (7.7%)	372 (65.1%)
	Not classifiable^b^	554	47 (8.5%)	177 (31.9%)
Median RDW, % (IQR)		13.8 (13.1–14.6)	14 (13.1–14.8)	14.1 (13.3–15.0)
	RDW>16%, n	188	17 (9%)	102 (54.3%)
	RDW ≤16%, n	1652	114 (6.5%)	600 (36.3%)
Median erythocyte count, T/l (IQR)		4.40 (4.00–4.70)	4.4 (4.1–4.7)	4.3 (3.9–4.6)
Median hematocrit, % (IQR)		38.9 (35.5–41.5)	39 (35.3–41.7)	37.9 (34.2–40.8)
Median hemoglobin concentration, g/dl (IQR)		12.8 (11.8–14.1)	13.1 (11.5–14.1)	12.7 (11.4–13.8)
Median MCV, fl (IQR)		88.6 (85.4–91.7)	87.7 (85.4–90.7)	88.4 (85.0–91.7)
Median MCH, pg (IQR)		29.9 (28.6–31.0)	29.9 (28.6–30.8)	29.9 (28.3–31.0)
Median MCHC, g/dl (IQR)		33.7 (32.9–34.4)	33.7 (33.1–34.5)	33.7 (32.8–34.3)
Use of ESA, n^c^		54	9 (16.7%)	38 (70.4%)

a Percentages are related to numbers given in the first column of the same line.

b Brain tumor, lymphoma and multiple myeloma.

c During the time period of one month before until 3 months after entry into the study.

*Abbreviations*: IQR = interquartile range (i.e. 25^th^–75^th^ percentile), RDW = red cell distribution width, MCV = mean corpuscular volume, MCH = mean corpuscular hemoglobin, MCHC = mean corpuscular hemoglobin concentration, ESA = erythropoiesis-stimulting agent.

### Thromboembolic events and death during the observation period

During a median follow-up time of 706 (95% CI: 653–730) days, 131 (7.1%) patients developed VTE and 702 (38.2%) died. Detailed characteristics of these patients are listed in table 1. Overall, 53 patients had an isolated DVT of the lower extremity, 53 an isolated PE, 7 a combined DVT of the lower extremity and PE; 4 patients each had a thrombosis of either the portal vein or the jugular vein; 6 patients had an isolated DVT of the upper extremity, and 1 patient each had a sinus vein thrombosis, combined DVT of the lower extremity and portal vein thrombosis, combined PE and DVT of the upper extremity or a thrombosis of the inferior caval vein. In 4 cases PE was fatal (3.1% of VTE events).

The cumulative probability of VTE in the total study population was 5.3% after 6 months, 6.5% after one year and 7.4% after two years. The cumulative probability of survival was 87.7% after 6 months, 74.2% after one year and 58% after two years.

### Distribution of RDW and other RBC parameters in the total study population

Median RDW (%) of the total study cohort was 13.8 (25^th^–75^th^ percentile: 13.1–14.6). RDW levels differed significantly between the various groups of different tumor types (p<0.001). The highest median RDW values were observed in patients with colorectal (14.2 [13.4–15.3]) and gastric cancer (14.2 [13.6–15.2]). Patients with solid tumors and distant metastasis had a significantly higher median RDW than those with localized solid tumors (14.1 [13.3–15.1] vs. 13.6 [13–14.2], p<0.001). Distribution of RDW and other RBC parameters in our study population is indicated in Table 1.

There was a weak to moderate correlation between RDW and the other RBC parameters measured by CBC: hemoglobin (r =  −0.41, p<0.001), erythrocyte count (r =  −0.18, p<0.001), hematocrit (r =  −0.33, p<0.001), mean corpuscular volume (MCV; r =  −0.23, p<0.001), mean corpuscular hemoglobin (MCH; r =  −0.40, p<0.001) and mean corpuscular hemoglobin concentration (MCHC; r =  −0.47, p<0.001). Furthermore, the correlation between RDW and CRP was analyzed. Data about CRP levels were available from 1533 study patients (83% of the total study population). A weak correlation between RDW and CRP was observed (r = 0.213, p<0.001).

In total, 188 patients (10.22% of the total study cohort) had a RDW above the upper reference range of our hospitals routine laboratory (>16%). The 188 patients with RDW>16% also had lower median hemoglobin levels, lower median hematocrit and a lower erythrocyte count compared to patients with normal RDW levels. Detailed numbers can be found in Table S1. Moreover, patients with RDW>16% were more frequently taking ESAs and iron supplementary therapy in comparison to patients who had lower RDW levels (ESAs: 20 [10.6%] vs. 34 [2.1%]; p<0.001; iron supplementary therapy: 12 [6.4%] vs. 12 [0.7%]; p<0.001).

Cancer patients had significantly higher RDW levels compared to healthy controls (median RDW: 13.8 [25^th^–75^th^ Percentile: 13.1–14.6] vs. 13.0 [12.5–13.4], p<0.001).

### RDW, other RBC parameters and risk of VTE in the total study cohort

The association between RDW or other RBC parameters and risk of VTE was calculated using proportional subdistribution hazards models (applying competing risk analysis) in order to adjust for death as a competing factor for occurrence of VTE. The univariable subhazard ratio (SHR) for occurrence of VTE was 1.03 (95% CI: 0.94–1.14, p = 0.478) per 1% increase in RDW.

**Figure 1:**
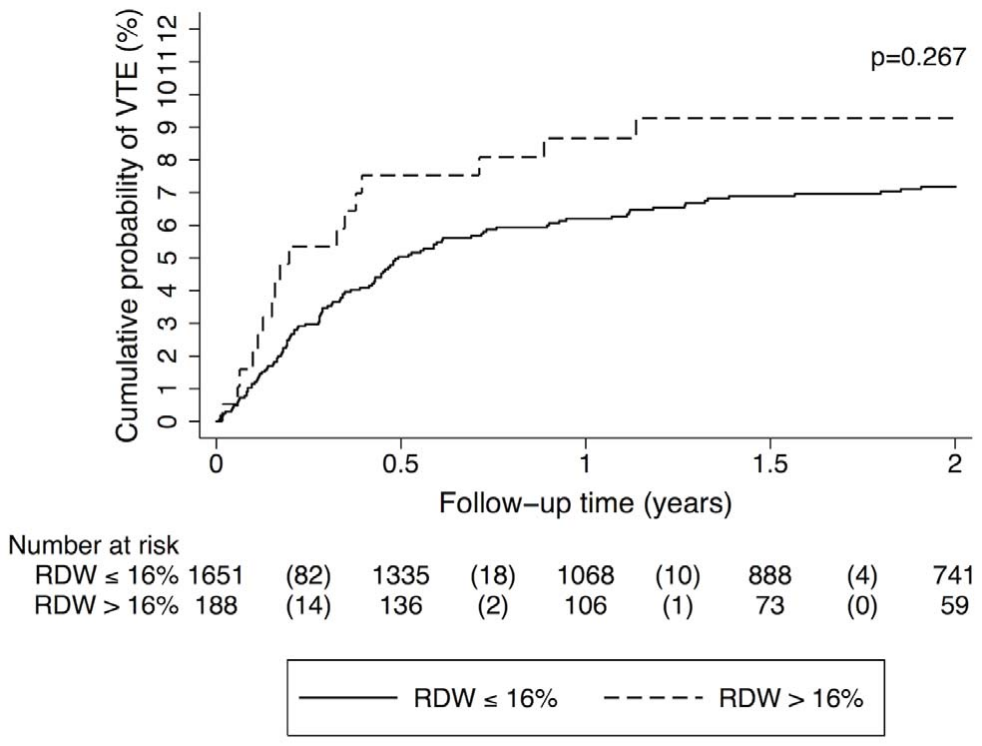
Cumulative incidence of venous thromboembolism (VTE), accounting for competing risk (death of any cause) in the total study cohort, grouped into patients with red cell distribution width (RDW)>16% and below (≤16%), respectively. In competing risk analysis (accounting for death of any cause), the probability of VTE was not significantly different between patients with high RDW (>16%) and patients with non-elevated RDW (Gray's test p = 0.267). Numbers in parentheses indicate numbers of VTE events in the respective group and time period.

Patients were divided into 2 groups to compare patients with high RDW (cut-off value was set at>16%, which is the upper limit of the reference range for RDW [reference range: 11%–16%] in our hospital's routine laboratory) to those with a lower RDW (≤16%). Patients with high RDW (n = 188, 10.2% of the total study population) had a similar risk of developing VTE as patients with lower RDW; the univariable SHR was 1.34 (95% CI: 0.80–2.23, p = 0.269). In different multivariable analyses, including the variables age and sex, tumor group (localized solid tumor vs. solid tumor with distant metastasis vs. non-classifiable [brain tumor or hematological malignancy]), use of ESA (during the time period of one month before until 3 months after entry into the study), hemoglobin level, leukocyte count, platelet count also no significant association between high RDW and increased risk of VTE was observed (data are shown in table 2). In patients with high RDW, the cumulative probability of VTE was 7.5% after 6 months, 8.7% after one year and 9.3% after two years in comparison to patients with a lower RDW with a probability of 5.0% after 6 months, 6.2% after one year and 7.2% after two years in those patients who had a lower RDW (Gray's test p = 0.267; Figure 1).

**Table 2:** Red blood cell parameters and risk of VTE in the total study cohort (n = 1840).

		N	Univariable HR (95% CI)	*p-value*	*Model 1* Multivariable HR (95% CI)	*p-value*	*Model 2* Multivariable HR (95% CI)	*p-value*	*Model 3*Multivariable HR (95% CI)	*p-value*	*Model 4* Multivariable HR (95% CI)	*p–value*
**RDW (%)**	**per 1% increase**	1840	1.03 (0.94–1.14)	*0.478*	1.04 (0.95–1.14)	*0.437*	1.02 (0.93–1.13)	*0.635*	1.02 (0.92–1.12)	*0.752*	1.04 (0.93–1.16)	*0.483*
	**RDW>16**	188	1.34 (0.80–2.23)	*0.269*	1.34 (0.80–2.24)	*0.261*	1.26 (0.74–2.13)	*0.389*	1.20 (0.71–2.05)	*0.50*	1.42 (0.81–2.49)	*0.227*
	**RDW ≤16**	1652	1.00 (Reference)	*N/A*	1.00 (Reference)	*N/A*	1.00 (Reference)	*N/A*	1.00 (Reference)	*N/A*	1.00 (Reference)	*N/A*
	**1^st^ quartile (<13.2)**	476	1.00 (Reference)	*N/A*	1.00 (Reference)	*N/A*	1.00 (Reference)	*N/A*	1.00 (Reference)	*N/A*	1.00 (Reference)	*N/A*
	**2^nd^ quartile (13.2–13.8)**	475	0.73 (0.44–1.21)	*0.217*	0.74 (0.44–1.23)	*0.242*	0.75 (0.45–1.24)	*0.260*	0.74 (0.45–1.23)	*0.249*	0.74 (0.44–1.22)	*0.237*
	**3^rd^ quartile (13.9–14.6)**	431	1.06 (0.66–1.70)	*0.801*	1.09 (0.68–1.74)	*0.733*	1.06 (0.66–1.70)	*0.802*	1.08 (0.67–1.73)	*0.757*	1.08 (0.67–1.75)	*0.742*
	**4^th^ quartile (>14.6)**	458	1.07 (0.67–1.70)	*0.790*	1.09 (0.68–1.75)	*0.725*	1.03 (0.64–1.64)	*0.915*	1.00 (0.62–1.63)	*0.922*	1.12 (0.67–1.87)	*0.678*
**Erythrocytes (T/l)**	**per 1 T/l increase**	1840	1.12 (0.82–1.55)	*0.477*	1.08 (0.78–1.49)	*0.657*	1.13 (0.82–1.57)	*0.460*	1.18 (0.83–1.66)	*0.357*	1.33 (0.82–2.16)	*0.242*
	**1^st^ quartile (<4.1)**	478	1.00 (Reference)	*N/A*	1.00 (Reference)	*N/A*	1.00 (Reference)	*N/A*	1.00 (Reference)	*N/A*	1.00 (Reference)	*N/A*
	**2^nd^ quartile (4.1–4.4)**	548	1.23 (0.77–1.97)	*0.378*	1.22 (0.76–1.95)	*0.405*	1.27 (0.79–2.04)	*0.315*	1.33 (0.81–2.17)	*0.254*	1.33 (0.79–2.23)	*0.285*
	**3^rd^ quartile (4.5–4.7)**	443	1.20 (0.73–1.96)	*0.472*	1.16 (0.70–1.91)	*0.560*	1.24 (0.74–2.06)	*0.417*	1.29 (0.76–2.20)	*0.338*	1.34 (0.75–2.39)	*0.316*
	**4^th^ quartile (>4.7)**	371	1.12 (0.66–1.89)	*0.680*	1.03 (0.60–1.78)	*0.902*	1.10 (0.64–1.92)	*0.723*	1.16 (0.65–2.06)	*0.613*	1.30 (0.66–2.53)	*0.445*
**Hematocrit (%)**	**per 1% increase**	1840	1.00 (0.97–1.04)	*0.821*	1.00 (0.97–1.04)	*1.000*	1.01 (0.97–1.04)	*0.728*	1.01 (0.98–1.05)	*0.555*	1.02 (0.97–1.06)	*0.443*
	**1^st^ quartile (<35.6)**	463	1.00 (Reference)	*N/A*	1.00 (Reference)	*N/A*	1.00 (Reference)	*N/A*	1.00 (Reference)	*N/A*	1.00 (Reference)	*N/A*
	**2^nd^ quartile (35.6–38.9)**	458	0.92 (0.57–1.50)	*0.744*	0.92 (0.56–1.50)	*0.729*	0.97 (0.59–1.59)	*0.893*	1.04 (0.61–1.76)	*0.884*	0.94 (0.50–1.76)	*0.843*
	**3^rd^ quartile (39.0–41.5)**	471	0.93 (0.57–1.51)	*0.771*	0.91 (0.56–1.48)	*0.710*	0.97 (0.59–1.60)	*0.913*	1.05 (0.61–1.79)	*0.860*	0.95 (0.44–2.04)	*0.902*
	**4^th^ quartile (>41.5)**	448	1.03 (0.64–1.66)	*0.903*	0.97 (0.60–1.57)	*0.900*	1.05 (0.64–1.74)	*0.837*	1.12 (0.66–1.89)	*0.683*	1.06 (0.42–2.67)	*0.895*
**Hemoglobin (g/dl)**	**per 1 g/dl increase**	1840	1.00 (0.90–1.11)	*0.989*	0.99 (0.89–1.09)	*0.811*	1.00 (0.91–1.11)	*0.941*	1.02 (0.92–1.14)	*0.711*	0.99 (0.90–1.10)	*0.898*
	**1^st^ quartile (<12.0)**	472	1.00 (Reference)	*N/A*	1.00 (Reference)	*N/A*	1.00 (Reference)	*N/A*	1.00 (Reference)	*N/A*	1.00 (Reference)	*N/A*
	**2^nd^ quartile (12.0–13.0)**	487	0.73 (0.45–1.18)	*0.199*	0.73 (0.45–1.17)	*0.191*	0.76 (0.47–1.25)	*0.284*	0.81 (0.49–1.33)	*0.403*	0.51 (0.25–1.02)	*0.056*
	**3^rd^ quartile (13.1–14.0)**	450	0.79 (0.49–1.28)	*0.341*	0.78 (0.48–1.25)	*0.300*	0.83 (0.51–1.35)	*0.444*	0.87 (0.53–1.45)	*0.600*	0.45 (0.18–1.14)	*0.092*
	**4^th^ quartile (>14.0)**	431	0.89 (0.56–1.42)	*0.626*	0.82 (0.51–1.34)	*0.435*	0.88 (0.53–1.46)	*0.627*	0.93 (0.55–1.56)	*0.772*	0.40 (0.11–1.39)	*0.148*
**MCV (fl)**	**per 1 fl increase**	1840	0.98 (0.96–1.00)	*0.052*	0.98 (0.97–1.00)	*0.064*	0.98 (0.96–1.00)	*0.058*	0.98 (0.97–1.00)	*0.110*	0.98 (0.96–1.00)	*0.039*
	**1^st^ quartile (<85.4)**	466	1.00 (Reference)	*N/A*	1.00 (Reference)	*N/A*	1.00 (Reference)	*N/A*	1.00 (Reference)	*N/A*	1.00 (Reference)	*N/A*
	**2^nd^ quartile (85.4–88.5)**	465	1.49 (0.96–2.32)	*0.079*	1.52 (0.97–2.38)	*0.067*	1.55 (0.99–2.42)	*0.056*	1.64 (1.05–2.57)	*0.030*	1.49 (0.96–2.29)	*0.073*
	**3^rd^ quartile (88.6–91.5)**	451	0.77 (0.46–1.30)	*0.329*	0.80 (0.47–1.35)	*0.397*	0.82 (0.48–1.38)	*0.445*	0.85 (0.50–1.43)	*0.539*	0.76 (0.45–1.29)	*0.310*
	**4^th^ quartile (>91.5)**	458	0.77 (0.45–1.29)	*0.313*	0.78 (0.46–1.32)	*0.356*	0.78 (0.46–1.31)	*0.349*	0.83 (0.49–1.40)	*0.480*	0.75 (0.45–1.25)	*0.270*
**MCH (pg)**	**per 1 pg increase**	1840	0.96 (0.90–1.03)	*0.235*	0.96 (0.90–1.02)	*0.221*	0.96 (0.90–1.03)	*0.243*	0.97 (0.91–1.04)	*0.368*	0.95 (0.88–1.02)	*0.178*
	**1^st^ quartile (<28.6)**	471	1.00 (Reference)	*N/A*	1.00 (Reference)	*N/A*	1.00 (Reference)	*N/A*	1.00 (Reference)	*N/A*	1.00 (Reference)	*N/A*
	**2^nd^ quartile (28.6–29.8)**	461	1.05 (0.65–1.69)	*0.848*	1.06 (0.65–1.72)	*0.812*	1.08 (0.66–1.76)	*0.749*	1.13 (0.70–1.84)	*0.622*	1.04 (0.65-1.66)	*0.870*
	**3^rd^ quartile (29.9–30.9)**	450	1.19 (0.75–1.90)	*0.457*	1.20 (0.75–1.91)	*0.453*	1.22 (0.77–1.95)	*0.398*	1.27 (0.80–2.04)	*0.31*	1.19 (0.74–1.90)	*0.477*
	**4^th^ quartile (>30.9)**	458	0.80 (0.48–1.34)	*0.392*	0.79 (0.47–1.32)	*0.371*	0.80 (0.48–1.34)	*0.402*	0.83 (0.50–1.40)	*0.493*	0.79 (0.47–1.33)	*0.374*
**MCHC (g/dl)**	**per 1 g/dl increase**	1840	1.04 (0.90–1.21)	*0.601*	1.03 (0.89–1.19)	*0.718*	1.03 (0.89–1.20)	*0.653*	1.06 (0.91–1.23)	*0.465*	1.07 (0.90–1.26)	*0.463*
	**1^st^ quartile (<32.9)**	496	1.00 (Reference)	*N/A*	1.00 (Reference)	*N/A*	1.00 (Reference)	*N/A*	1.00 (Reference)	*N/A*	1.00 (Reference)	*N/A*
	**2^nd^ quartile (32.9–33.6)**	491	1.17 (0.72–1.88)	*0.530*	1.17 (0.72–1.89)	*0.533*	1.20 (0.73–1.95)	*0.471*	1.23 (0.75–2.01)	*0.405*	1.24 (0.76–2.03)	*0.386*
	**3^rd^ quartile (33.7–34.3)**	432	1.08 (0.65–1.79)	*0.780*	1.05 (0.63–1.75)	*0.855*	1.07 (0.64–1.79)	*0.805*	1.15 (0.68–1.94)	*0.604*	1.17 (0.68–2.01)	*0.576*
	**4^th^ quartile (>34.3)**	421	1.33 (0.82–2.15)	*0.249*	1.27 (0.79–2.07)	*0.326*	1.29 (0.80–2.10)	*0.297*	1.37 (0.84–2.23)	*0.214*	1.47 (0.87–2.51)	*0.154*

*Model 1:* adjusted for age and sex;

*Model 2:* adjusted for age, sex and tumor group (localized solid tumor vs. solid tumor with distant metastasis vs. non-classifiable [brain tumor or hematological malignancy]);

*Model 3:* adjusted for age, sex and use of ESA;

*Model 4:* adjusted for age, sex, hemoglobin level, leukocyte count and platelet count.

*Abbreviations*: SHR = subhazard ratio, CI = confidence interval, RDW = red cell distribution width, MCV = mean corpuscular volume, MCH = mean corpuscular hemoglobin, MCHC = mean corpuscular hemoglobin concentration, ESA = erythropoiesis-stimulating agent.

Furthermore, also the association between other RBC parameters (erythrocyte count, hematocrit, hemoglobin, MCV, MCH, MCHC) and risk of VTE was analyzed. We did not observe a significant association between these parameters and risk of VTE, neither when analyzed as continuous variables nor when analyzed as categorized variables according to quartiles of the respective parameter in the total study population (Table 2). Since low hemoglobin was found to be a risk factor for VTE in previous studies and was incorporated into the Khorana-Score [15], we divided patients into two groups, according to their hemoglobin levels (based on the Khorana-Score the cut-off was set at <10 g/dl). Also in this analysis, hemoglobin levels were not associated with risk of VTE (data are not shown, but can be provided upon request).

### RDW, other RBC parameters and risk of VTE in subgroups of cancer patients

**Figure 2:**
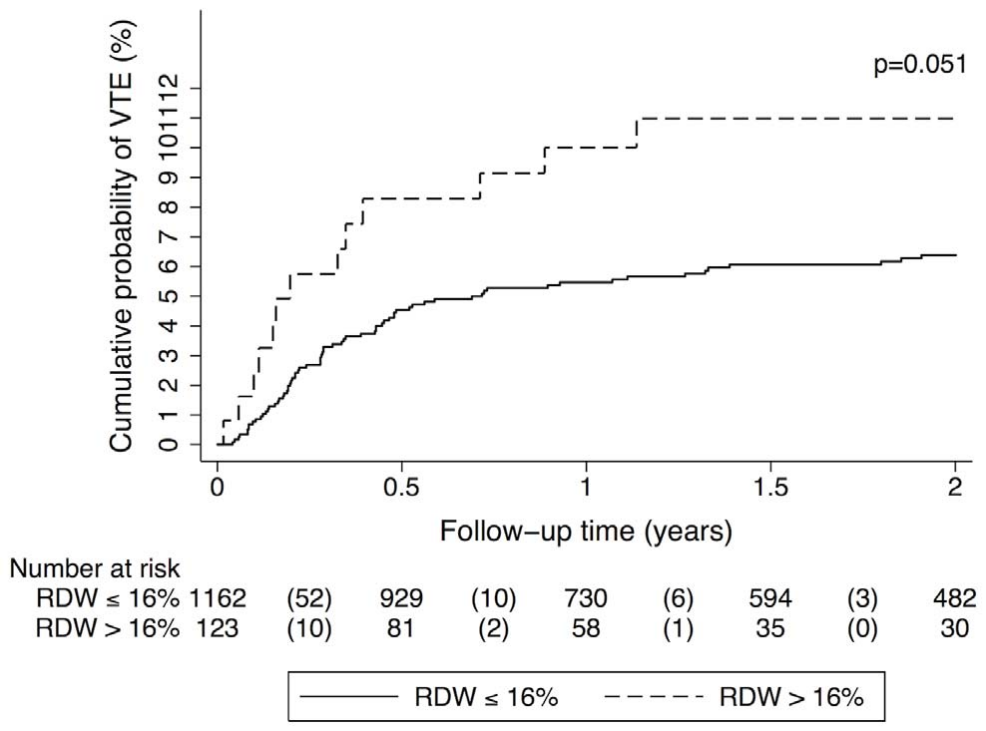
Cumulative incidence of venous thromboembolism (VTE), accounting for competing risk (death of any cause) in patients with solid tumors, grouped into patients with red cell distribution width (RDW)>16% and below (≤16%), respectively. The probability of VTE was higher in patients with high RDW (>16%) compared to patients with lower RDW (Gray's test p = 0.051). Numbers in parentheses indicate numbers of VTE events in the respective group and time period.

As our study population consists of a very heterogeneous group of patients with different cancer sites, including hematological malignancies or brain tumors, we conducted a subgroup analysis of patients with solid tumors only. In this subgroup high RDW was associated with an 80% increase in risk of VTE compared to lower RDW (SHR [95% CI]: 1.80 [0.99–3.26], p = 0.053). However, in multivariable analyses (adjusting for the same variables as in our analyses conducted in the total study cohort) we did not observe a significant association between RDW and risk of VTE (data are shown in table 3). The cumulative probability of VTE in patients with high RDW was 8.3% after 6 months, 10.0% after one year and 11.0% after two years in comparison to 4.5%, 5.5% and 6.4%, respectively in those patients who had a lower RDW (Gray's test p = 0.051; Figure 2).

**Table 3:** Red blood cell parameters and risk of VTE in patients with solid tumors (n = 1286). Significant results are highlighted in bold.

		N	Univariable HR (95% CI)	*p–value*	*Model 1* Multivariable HR (95% CI)	*p-value*	*Model 2*Multivariable HR (95% CI)	*p-value*	*Model 3*Multivariable HR (95% CI)	*p-value*	*Model 4*Multivariable HR (95% CI)	*p-value*
**RDW (%)**	**per 1% increase**	1286	1.07 (0.96–1.19)	*0.250*	1.07 (0.96–1.19)	*0.228*	1.06 (0.94–1.18)	*0.356*	1.04 (0.93–1.16)	*0.522*	1.02 (0.89–1.17)	*0.766*
	**RDW>16**	123	**1.80 (0.99–3.26)**	***0.053***	**1.82 (0.99–3.32)**	***0.053***	1.70 (0.91–3.19)	*0.097*	1.57 (0.83–2.94)	*0.164*	1.57 (0.81–3.04)	*0.182*
	**RDW ≤16**	1163	1.00 (Reference)	*N/A*	1.00 (Reference)	*N/A*	1.00 (Reference)	*N/A*	1.00 (Reference)	*N/A*	1.00 (Reference)	*N/A*
	**1^st^ quartile (<13.2)**	328	1.00 (Reference)	*N/A*	1.00 (Reference)	*N/A*	1.00 (Reference)	*N/A*	1.00 (Reference)	*N/A*	1.00 (Reference)	*N/A*
	**2^nd^ quartile (13.2–13.7)**	316	0.52 (0.27–1.02)	*0.056*	0.53 (0.27–1.03)	*0.060*	0.52 (0.27–1.02)	*0.056*	0.51 (0.26–0.97)	*0.040*	0.52 (0.27–1.02)	*0.058*
	**3^rd^ quartile (13.8–14.5)**	321	0.84 (0.47–1.51)	*0.564*	0.86 (0.48–1.52)	*0.597*	0.84 (0.47–1.49)	*0.553*	0.94 (0.53–1.66)	*0.821*	0.82 (0.46–1.46)	*0.497*
	**4^th^ quartile (>14.5)**	321	1.01 (0.58–1.75)	*0.978*	1.02 (0.58–1.79)	*0.934*	0.95 (0.54–1.67)	*0.865*	0.88 (0.48–1.62)	*0.687*	0.85 (0.46–1.56)	*0.599*
**Erythrocytes (T/l)**	**per 1 T/l increase**	1286	0.97 (0.61–1.53)	*0.880*	0.91 (0.58–1.44)	*0.696*	0.97 (0.61–1.54)	*0.891*	1.03 (0.63–1.68)	*0.910*	1.49 (0.77–2.87)	*0.237*
	**1^st^ quartile (<4.2)**	415	1.00 (Reference)	*N/A*	1.00 (Reference)	*N/A*	1.00 (Reference)	*N/A*	1.00 (Reference)	*N/A*	1.00 (Reference)	*N/A*
	**2^nd^ quartile (4.2–4.4)**	311	1.52 (0.88–2.64)	*0.132*	1.51 (0.87–2.61)	*0.144*	1.54 (0.89–2.67)	*0.125*	1.44 (0.80–2.60)	*0.226*	1.95 (1.08–3.51)	*0.026*
	**3^rd^ quartile (4.5–4.7)**	314	0.99 (0.54–1.82)	*0.966*	0.95 (0.52–1.75)	*0.879*	1.01 (0.54–1.87)	*0.985*	1.05 (0.53–2.08)	*0.880*	1.44 (0.75–2.78)	*0.273*
	**4^th^ quartile (>4.7)**	246	1.04 (0.55–1.99)	*0.898*	0.95 (0.49–1.84)	*0.889*	1.02 (0.52–1.99)	*0.958*	1.06 (0.51–2.21)	*0.868*	1.78 (0.82–3.85)	*0.144*
**Hematocrit (%)**	**per 1% increase**	1286	0.97 (0.93–1.02)	*0.308*	0.97 (0.93–1.02)	*0.228*	0.98 (0.93–1.03)	*0.381*	0.99 (0.94–1.04)	*0.591*	1.02 (0.98–1.07)	*0.337*
	**1^st^ quartile (<35.8)**	331	1.00 (Reference)	*N/A*	1.00 (Reference)	*N/A*	1.00 (Reference)	*N/A*	1.00 (Reference)	*N/A*	1.00 (Reference)	*N/A*
	**2^nd^ quartile (35.8–38.9)**	323	0.61 (0.34–1.12)	*0.112*	0.61 (0.33–1.12)	*0.111*	0.63 (0.34-1.17)	*0.141*	0.75 (0.39–1.45)	*0.395*	0.71 (0.33–1.54)	*0.392*
	**3^rd^ quartile (39.0–41.4)**	311	0.75 (0.43–1.34)	*0.337*	0.74 (0.42–1.33)	*0.319*	0.79 (0.43–1.44)	*0.445*	0.85 (0.44–1.65)	*0.628*	0.94 (0.37–2.37)	*0.899*
	**4^th^ quartile (>41.4)**	321	0.68 (0.38–1.22)	*0.195*	0.64 (0.36–1.14)	*0.128*	0.69 (0.38–1.27)	*0.233*	0.81 (0.42–1.55)	*0.519*	0.94 (0.31–2.83)	*0.905*
**Hemoglobin (g/dl)**	**per 1 g/dl increase**	1286	0.91 (0.80–1.04)	*0.165*	0.90 (0.79–1.02)	*0.103*	0.91 (0.80–1.05)	*0.198*	0.94 (0.81–1.08)	*0.390*	0.92 (0.81–1.05)	*0.228*
	**1^st^ quartile (<11.9)**	343	1.00 (Reference)	*N/A*	1.00 (Reference)	*N/A*	1.00 (Reference)	*N/A*	1.00 (Reference)	*N/A*	1.00 (Reference)	*N/A*
	**2^nd^ quartile (11.9–13.1)**	319	**0.54 (0.30–0.99)**	***0.047***	**0.54 (0.29–0.99)**	***0.047***	0.55 (0.30–1.02)	*0.058*	0.69 (0.37–1.29)	*0.247*	0.53 (0.23–1.21)	*0.129*
	**3^rd^ quartile (13.2–14.1)**	313	0.62 (0.34–1.10)	*0.101*	0.59 (0.33–1.07)	*0.083*	0.63 (0.34–1.16)	*0.139*	0.72 (0.38–1.37)	*0.321*	0.58 (0.21–1.62)	*0.299*
	**4^th^ quartile (>14.1)**	311	0.66 (0.37–1.16)	*0.147*	0.61 (0.35–1.08)	*0.090*	0.66 (0.36–1.18)	*0.162*	0.73 (0.37–1.42)	*0.352*	0.60 (0.15–2.34)	*0.463*
**MCV (fl)**	**per 1 fl increase**	1286	**0.98 (0.96–1.00)**	***0.022***	**0.98 (0.96–1.00)**	***0.032***	**0.98 (0.96–1.00)**	***0.040***	0.98 (0.96–1.00)	*0.078*	0.98 (0.96–1.01)	*0.140*
	**1^st^ quartile (<85.5)**	323	1.00 (Reference)	*N/A*	1.00 (Reference)	*N/A*	1.00 (Reference)	*N/A*	1.00 (Reference)	*N/A*	1.00 (Reference)	*N/A*
	**2^nd^ quartile (85.5–88.6)**	330	1.10 (0.64–1.88)	*0.732*	1.12 (0.65–1.94)	*0.680*	1.15 (0.67–1.99)	*0.609*	1.26 (0.74–2.17)	*0.396*	1.20 (0.71–2.03)	*0.491*
	**3^rd^ quartile (88.7–91.7)**	316	0.72 (0.39–1.32)	*0.290*	0.75 (0.40–1.39)	*0.359*	0.79 (0.43–1.47)	*0.455*	0.71 (0.37–1.33)	*0.284*	0.80 (0.43–1.49)	*0.484*
	**4^th^ quartile (>91.7)**	317	0.52 (0.27–1.02)	*0.056*	0.54 (0.27–1.05)	*0.071*	0.55 (0.28–1.08)	*0.083*	0.60 (0.30–1.20)	*0.149*	0.59 (0.30–1.16)	*0.128*
**MCH (pg)**	**per 1 pg increase**	1286	**0.92 (0.85–1.00)**	***0.044***	**0.92 (0.85–1.00)**	***0.047***	0.93 (0.85–1.01)	*0.072*	0.94 (0.86–1.02)	*0.152*	0.95 (0.86–1.04)	*0.260*
	**1^st^ quartile (<28.7)**	336	1.00 (Reference)	*N/A*	1.00 (Reference)	*N/A*	1.00 (Reference)	*N/A*	1.00 (Reference)	*N/A*	1.00 (Reference)	*N/A*
	**2^nd^ quartile (28.8–29.9)**	317	0.91 (0.52–1.62)	*0.759*	0.93 (0.52–1.66)	*0.807*	0.97 (0.54–1.75)	*0.916*	1.05 (0.59–1.89)	*0.864*	1.04 (0.59–1.84)	*0.899*
	**3^rd^ quartile (30.0–31.0)**	317	1.04 (0.60–1.81)	*0.894*	1.05 (0.60–1.83)	*0.870*	1.10 (0.63–1.94)	*0.735*	1.14 (0.64–2.01)	*0.656*	1.21 (0.66–2.22)	*0.530*
	**4^th^ quartile (>31.0)**	316	**0.49 (0.25–0.99)**	***0.046***	**0.49 (0.25–0.99)**	***0.046***	0.52 (0.26–1.05)	*0.067*	0.54 (0.26–1.12)	*0.097*	0.60 (0.28–1.26)	*0.177*
**MCHC (g/dl)**	**per 1 g/dl increase**	1286	0.92 (0.76–1.12)	*0.408*	0.91 (0.75–1.10)	*0.319*	0.92 (0.76–1.11)	*0.403*	0.96 (0.79–1.17)	*0.683*	1.01 (0.81–1.25)	*0.949*
	**1^st^ quartile (<33.0)**	332	1.00 (Reference)	*N/A*	1.00 (Reference)	*N/A*	1.00 (Reference)	*N/A*	1.00 (Reference)	*N/A*	1.00 (Reference)	*N/A*
	**2^nd^ quartile (33.0–33.7)**	331	1.04 (0.57–1.89)	*0.889*	1.05 (0.57–1.93)	*0.869*	1.10 (0.59–2.03)	*0.765*	1.28 (0.70–2.31)	*0.421*	1.30 (0.71–2.39)	*0.399*
	**3^rd^ quartile (33.8–34.4)**	322	1.13 (0.62–2.04)	*0.691*	1.11 (0.61–2.02)	*0.743*	1.15 (0.62–2.11)	*0.661*	1.18 (0.62–2.25)	*0.610*	1.49 (0.78–2.85)	*0.225*
	**4^th^ quartile (>34.4)**	301	0.93 (0.49–1.74)	*0.816*	0.88 (0.47–1.66)	*0.701*	0.92 (0.49–1.73)	*0.800*	1.04 (0.53–2.04)	*0.902*	1.31 (0.65–2.63)	*0.445*

*Model 1:* adjusted for age and sex;

*Model 2:* adjusted for age, sex and tumor group (localized solid tumor vs. solid tumor with distant metastasis vs. non-classifiable [brain tumor or hematological malignancy]);

*Model 3:* adjusted for age, sex and use of ESA;

*Model 4:* adjusted for age, sex, hemoglobin level, leukocyte count and platelet count.

*Abbreviations*: SHR = subhazard ratio, CI = confidence interval, RDW = red cell distribution width, MCV = mean corpuscular volume, MCH = mean corpuscular hemoglobin, MCHC = mean corpuscular hemoglobin concentration, ESA = erythropoiesis-stimulating agent.

Increasing levels of hemoglobin, MCV and MCH were associated with a reduced risk of VTE in univariable and also in some multivariable analyses. However, these associations did not remain statistically significant after adjustment for tumor group or other CBC parameters (hemoglobin, leukocyte count, platelet count) in multivariable analyses (Table 3). We did not observe a significant association of erythrocyte count, hematocrit or MCHC with the risk of VTE, neither when analyzed as continuous variables nor when analyzed as categorized variables according to quartiles of the respective parameter (Table 3).

Furthermore, we analyzed RDW and other RBC parameters in subgroups of patients with different tumor sites (brain, breast, lung, colon, pancreas or lymphoma [analyses of other tumor sites was not possible due to the limited number of patients and/or thrombotic events in these subgroups, respectively]). There was no significant association between RBC parameters and the risk of VTE in these subgroups (data are not shown, but can be provided upon request).

### RDW, other RBC parameters and risk of mortality in patients with cancer

Increasing RDW was associated with a significantly increased risk of mortality; the hazard ratio (HR) per 1% increase of RDW was 1.11 (95% CI: 1.08–1.15, p<0.001). For further analyses, patients were again divided into two groups to compare patients with high RDW (>16%) to those with non-elevated RDW (≤16%). High RDW was associated with an increased risk of mortality (HR [95% CI]: 1.72 [1.39–2.12], p<0.001). In multivariable analyses, adjusted for age, sex, tumor group (localized solid tumor vs. solid tumor with distant metastasis vs. non-classifiable [brain tumor or hematological malignancy]), use of ESA, hemoglobin level, leukocyte count or platelet count, the impact of RDW on mortality was attenuated. However, in all multivariable models higher RDW was associated with a significantly increased risk of mortality (Table 4).

**Table 4:** Red blood cell parameters and risk of mortality in the total study cohort (n = 1840). Significant results are highlighted in bold.

		N	Univariable HR (95% CI)	*p-value*	*Model 1* Multivariable HR (95% CI)	*p-value*	*Model 2*Multivariable HR (95% CI)	*p-value*	*Model 3*Multivariable HR (95% CI)	*p-value*	*Model 4*Multivariable HR (95% CI)	*p-value*
**RDW (%)**	**per 1% increase**	1840	**1.11 (1.08–1.15)**	***<0.001***	**1.10 (1.06–1.14**)	***<0.001***	**1.05 (1.01–1.09)**	***0.018***	**1.08 (1.04–1.13)**	***<0.001***	1.04 (0.99–1.08)	0.097
	**RDW>16**	188	**1.72 (1.39–2.12)**	***<0.001***	**1.59 (1.29–1.97)**	***<0.001***	**1.27 (1.03–1.58)**	***0.027***	**1.51 (1.22–1.87)**	***<0.001***	**1.34 (1.06–1.70)**	**0.016**
	**RDW ≤16**	1652	1.00 (Reference)	*N/A*	1.00 (Reference)	*N/A*	1.00 (Reference)	*N/A*	1.00 (Reference)	*N/A*	1.00 (Reference)	N/A
	**1^st^ quartile (<13.2)**	476	1.00 (Reference)	*N/A*	1.00 (Reference)	*N/A*	1.00 (Reference)	*N/A*	1.00 (Reference)	*N/A*	1.00 (Reference)	N/A
	**2^nd^ quartile (13.2–13.8)**	475	0.99 (0.79–1.25)	*0.950*	0.95 (0.75–1.20)	*0.654*	0.90 (0.71–1.13)	*0.360*	0.95 (0.76–1.20)	*0.685*	0.97 (0.77–1.23)	0.827
	**3^rd^ quartile (13.9–14.6)**	431	**1.61 (1.29–2.00)**	***<0.001***	**1.48 (1.19–1.84)**	***0.001***	**1.32 (1.06–1.65)**	***0.013***	**1.47 (1.18–1.83)**	***0.001***	**1.55 (1.24–1.93)**	**<0.001**
	**4^th^ quartile (>14.6)**	458	**1.90 (1.54–2.35)**	***<0.001***	**1.72 (1.39–2.13)**	***<0.001***	**1.36 (1.09–1.69)**	***0.006***	**1.65 (1.33–2.05)**	***<0.001***	**1.62 (1.28–2.05)**	**<0.001**
**Erythrocytes (T/l)**	**per 1 T/l increase**	1840	**0.66 (0.57–0.75)**	***<0.001***	**0.66 (0.58–0.75)**	***<0.001***	**0.70 (0.61–0.81)**	***<0.001***	**0.69 (0.60–0.79)**	***<0.001***	**0.75 (0.60–0.95)**	**0.015**
	**1^st^ quartile (<4.1)**	478	1.00 (Reference)	*N/A*	1.00 (Reference)	*N/A*	1.00 (Reference)	*N/A*	1.00 (Reference)	*N/A*	1.00 (Reference)	N/A
	**2^nd^ quartile (4.1–4.4)**	548	**0.76 (0.63–0.91)**	***0.004***	**0.78 (0.65–0.94)**	***0.009***	**0.80 (0.67–0.97)**	***0.022***	**0.80 (0.67–0.97)**	***0.023***	0.85 (0.69–1.05)	0.131
	**3^rd^ quartile (4.5–4.7)**	443	**0.55 (0.45–0.68)**	***<0.001***	**0.56 (0.45–0.69)**	***<0.001***	**0.63 (0.51–0.78)**	***<0.001***	**0.58 (0.47–0.72)**	***<0.001***	**0.67 (0.52–0.88)**	**0.004**
	**4^th^ quartile (>4.7)**	371	**0.58 (0.46–0.72)**	***<0.001***	**0.57 (0.45–0.71)**	***<0.001***	**0.65 (0.52–0.82)**	***<0.001***	**0.59 (0.47–0.74)**	***<0.001***	0.77 (0.56–1.04)	0.090
**Hematocrit (%)**	**per 1% increase**	1840	**0.95 (0.94–0.96)**	***<0.001***	**0.95 (0.94–0.96)**	***<0.001***	**0.96 (0.95–0.98)**	***<0.001***	**0.95 (0.94–0.97)**	***<0.001***	**0.96 (0.93–0.99)**	**0.015**
	**1^st^ quartile (<35.6)**	463	1.00 (Reference)	*N/A*	1.00 (Reference)	*N/A*	1.00 (Reference)	*N/A*	1.00 (Reference)	*N/A*	1.00 (Reference)	N/A
	**2^nd^ quartile (35.6–38.9)**	458	**0.82 (0.67–0.99)**	***0.038***	0.86 (0.71–1.04)	*0.127*	0.89 (0.73–1.08)	*0.230*	0.91 (0.74–1.10)	*0.323*	0.85 (0.66–1.11)	0.230
	**3^rd^ quartile (39.0–41.5)**	471	**0.55 (0.45–0.68)**	***<0.001***	**0.57 (0.47–0.71)**	***<0.001***	**0.70 (0.56–0.86)**	***0.001***	**0.61 (0.49–0.75)**	***<0.001***	**0.60 (0.43–0.83)**	**0.002**
	**4^th^ quartile (>41.5)**	448	**0.52 (0.42–0.64)**	***<0.001***	**0.50 (0.41–0.63)**	***<0.001***	**0.62 (0.50–0.78)**	***<0.001***	**0.53 (0.43–0.66)**	***<0.001***	**0.58 (0.38–0.89)**	**0.012**
**Hemoglobin (g/dl)**	**per 1 g/dl increase**	1840	**0.88 (0.84–0.91)**	***<0.001***	**0.88 (0.84–0.91)**	***<0.001***	**0.91 (0.87–0.95)**	***<0.001***	**0.89 (0.85–0.92)**	***<0.001***	**0.88 (0.85–0.92)**	**<0.001**
	**1^st^ quartile (<12.0)**	472	1.00 (Reference)	*N/A*	1.00 (Reference)	*N/A*	1.00 (Reference)	*N/A*	1.00 (Reference)	*N/A*	1.00 (Reference)	N/A
	**2^nd^ quartile (12.0–13.0)**	487	0.79 (0.65–0.95)	*0.013*	0.84 (0.70–1.02)	*0.077*	0.88 (0.72–1.06)	*0.175*	0.89 (0.73–1.08)	*0.236*	0.85 (0.64–1.12)	0.244
	**3^rd^ quartile (13.1–14.0)**	450	**0.51 (0.41–0.63)**	***<0.001***	**0.53 (0.43–0.66)**	***<0.001***	**0.65 (0.52–0.80)**	***<0.001***	**0.56 (0.45–0.70)**	***<0.001***	**0.58 (0.39–0.84)**	**0.005**
	**4^th^ quartile (>14.0)**	431	**0.55 (0.45–0.68)**	***<0.001***	**0.54 (0.43–0.67)**	***<0.001***	**0.70 (0.56–0.87)**	***0.002***	**0.57 (0.46–0.71)**	***<0.001***	0.66 (0.40–1.07)	0.094
**MCV (fl)**	**per 1 fl increase**	1840	1.00 (0.99–1.01)	*0.525*	0.99 (0.98–1.01)	*0.303*	1.00 (0.99–1.01)	*0.900*	1.00 (0.99–1.01)	*0.446*	1.01 (1.00–1.02)	0.162
	**1^st^ quartile (<85.4)**	466	1.00 (Reference)	*N/A*	1.00 (Reference)	*N/A*	1.00 (Reference)	*N/A*	1.00 (Reference)	*N/A*	1.00 (Reference)	N/A
	**2^nd^ quartile (85.4–88.5)**	465	0.94 (0.76–1.15)	*0.529*	0.91 (0.74–1.12)	*0.390*	1.04 (0.84–1.27)	*0.731*	0.96 (0.78–1.19)	*0.718*	1.13 (0.91–1.40)	0.265
	**3^rd^ quartile (88.6–91.5)**	451	0.82 (0.67–1.02)	*0.072*	0.79 (0.64–0.98)	*0.032*	0.96 (0.77–1.18)	*0.680*	0.83 (0.67–1.02)	*0.082*	1.00 (0.80–1.24)	0.981
	**4^th^ quartile (>91.5)**	458	0.90 (0.73–1.11)	*0.320*	0.85 (0.69–1.05)	*0.133*	0.97 (0.79–1.19)	*0.752*	0.88 (0.71–1.08)	*0.222*	1.08 (0.87–1.34)	0.489
**MCH (pg)**	**per 1 pg increase**	1840	0.97 (0.95–1.00)	*0.062*	**0.97 (0.94–1.00)**	***0.044***	1.00 (0.97–1.03)	*0.996*	0.98 (0.95–1.01)	*0.109*	1.02 (1.00–1.04)	0.094
	**1^st^ quartile (<28.6)**	471	1.00 (Reference)	*N/A*	1.00 (Reference)	*N/A*	1.00 (Reference)	*N/A*	1.00 (Reference)	*N/A*	1.00 (Reference)	N/A
	**2^nd^ quartile (28.6–29.8)**	461	**0.77 (0.62–0.94)**	***0.011***	**0.78 (0.63–0.96)**	***0.019***	0.91 (0.74–1.12)	*0.395*	0.80 (0.65–0.99)	*0.040*	0.95 (0.76–1.18)	0.641
	**3^rd^ quartile (29.9–30.9)**	450	**0.78 (0.64–0.96)**	***0.018***	**0.77 (0.63–0.95)**	***0.013***	0.93 (0.75–1.14)	*0.459*	**0.79 (0.64–0.97)**	***0.026***	1.03 (0.82–1.29)	0.783
	**4^th^ quartile (>30.9)**	458	**0.79 (0.64–0.96)**	***0.020***	**0.77 (0.63–0.94)**	***0.012***	0.96 (0.78–1.18)	*0.675*	**0.79 (0.64–0.97)**	***0.023***	1.04 (0.83–1.30)	0.723
**MCHC (g/dl)**	**per 1 g/dl increase**	1840	0.95 (0.90–1.01)	*0.122*	0.96 (0.91–1.02)	*0.227*	1.02 (0.96–1.08)	*0.528*	0.98 (0.92–1.04)	*0.441*	**1.14 (1.06–1.22)**	**<0.001**
	**1^st^ quartile (<32.9)**	496	1.00 (Reference)	*N/A*	1.00 (Reference)	*N/A*	1.00 (Reference)	*N/A*	1.00 (Reference)	*N/A*	1.00 (Reference)	N/A
	**2^nd^ quartile (32.9–33.6)**	491	**0.80 (0.65–0.98)**	***0.029***	**0.82 (0.67–1.00)**	***0.050***	0.92 (0.75–1.12)	*0.393*	0.83 (0.68–1.02)	*0.078*	1.06 (0.85–1.31)	0.628
	**3^rd^ quartile (33.7–34.3)**	432	0.88 (0.72–1.08)	*0.221*	0.91 (0.74–1.12)	*0.369*	1.07 (0.87–1.31)	*0.531*	0.95 (0.77–1.17)	*0.630*	**1.26 (1.00–1.59)**	**0.051**
	**4^th^ quartile (>34.3)**	421	**0.81 (0.65–0.99)**	***0.044***	0.82 (0.66–1.01)	*0.062*	0.97 (0.78–1.20)	*0.778*	0.84 (0.68–1.04)	*0.111*	1.24 (0.97–1.59)	0.083

*Model 1:* adjusted for age and sex;

*Model 2:* adjusted for age, sex and tumor group (localized solid tumor vs. solid tumor with distant metastasis vs. non-classifiable [brain tumor or hematological malignancy]);

*Model 3:* adjusted for age, sex and use of ESA;

*Model 4:* adjusted for age, sex, hemoglobin level, leukocyte count and platelet count.

*Abbreviations*: SHR = subhazard ratio, CI = confidence interval, RDW = red cell distribution width, MCV = mean corpuscular volume, MCH = mean corpuscular hemoglobin, MCHC = mean corpuscular hemoglobin concentration, ESA = erythropoiesis-stimulating agent.

**Figure 3:**
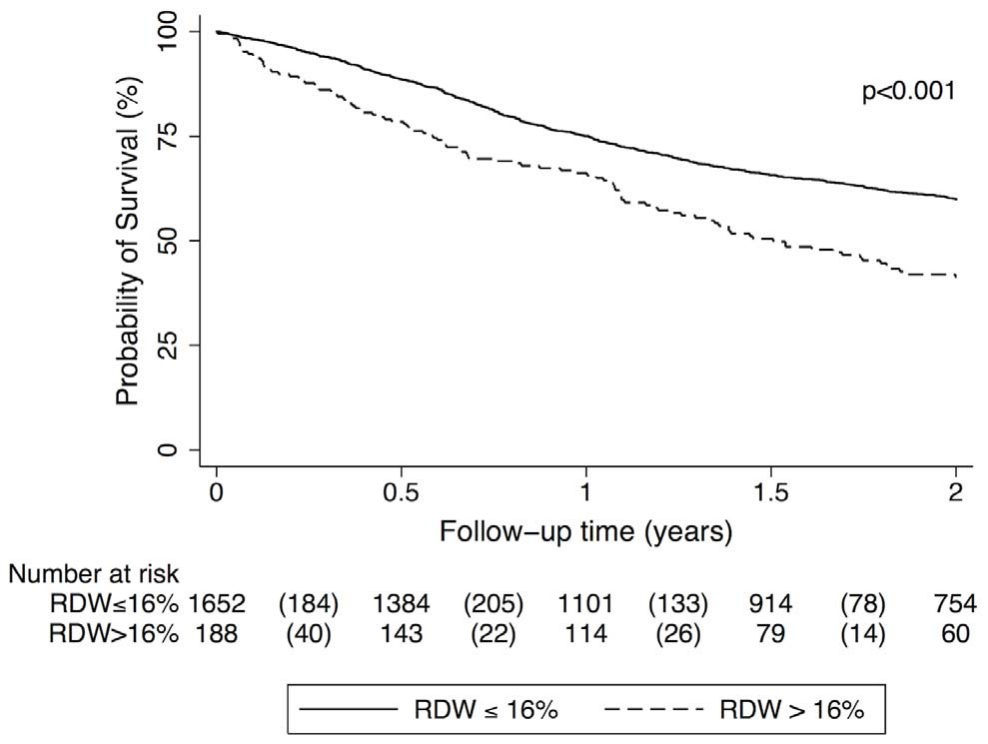
Kaplan-Meier estimates for cumulative survival probability of cancer patients (total study cohort) with red blood cell distribution width (RDW)>16% and below (≤16%), respectively. Survival rates were significantly lower in patients with high RDW in comparison to those with a non-elevated RDW (p<0.001). Numbers in parentheses indicate numbers of deaths in the respective group and time period.

The cumulative probability of survival in patients with high RDW (>16%) was 78.5% after 6 months, 66.2% after one year and 41.3% after two years. In comparison, patients with non-elevated RDW levels had a cumulative survival probability of 88.7% after 6 months, 75.1% after one year and 66.2% after two years (Log-rank p<0.001; Figure 3).

Associations of other RBC parameters with risk of mortality in the total study cohort are shown in table 4. Higher levels of erythrocyte counts, hematocrit, hemoglobin, MCH and MCHC were associated with a reduced risk of mortality (Table 4).

## Discussion

In this prospective, observational cohort study of patients with various types of cancer we did not observe an independent association between RDW or other RBC parameters (erythrocyte count, hematocrit, hemoglobin, MCV, MCH, MCHC) and the risk of future VTE. As our study population consists of a very heterogeneous group of cancer patients, including patients with hematological malignancies and patients with malignant brain tumors, we conducted a subgroup analysis of solid tumors only. In this patient group, elevated RDW (>16%) was associated with an 80% higher risk of VTE compared to patients with non-elevated RDW. However, this association was only borderline significant and did not prevail after adjustment for age, sex, tumor group, use of ESA or other parameters of the CBC. Furthermore, higher levels of hemoglobin, MCV and MCH were associated with a reduced risk of VTE in patients with solid tumors in some analyses; however, none of these associations remained statistically significant in multivariable analyses.

Our findings are in contrast to previous studies, which had shown that high RDW [8] and also high hematocrit [13] are associated with an increased risk of VTE in the general population. However, our results might in fact not be conflicting after all, since our study cohort consists of patients with cancer and these patients were either excluded [8] or under-represented (only 23% of VTE events were related to cancer) in the aforementioned studies [13]. Although reported in previous studies [15], we did not observe an association between levels of hemoglobin and risk of cancer-associated VTE. A low hemoglobin level was incorporated in the Khorana-Score for risk assessment of VTE in patients with cancer [15]. However, in this score one point is given for the presence of low hemoglobin levels and/or the use of ESA; and the use of ESA is known to lead to a high thrombotic risk [22], [23]. ESA were only very rarely used in our study population (only 2.9% of study patients were using ESA), in contrast to the cohorts on whom the Khorana-Score was established (in these cohorts almost one third of patients received ESA), which might in part explain the discrepancies between the studies. In our study adjustment for use of ESA did not influence the association of RBC parameters with VTE risk.

RBCs are major constituents of venous blood clots [24] and experimental data indicate that they are not only inactive, random cell material trapped during clotting but might also actively participate in thrombus formation [25], [26]. RBC have an impact on blood viscosity, they release pro-coagulatory factors and they can expose phosphatidylserine on their surface and thereby promote activation of coagulation [25]. However, whether there is a direct biological link between RBC and VTE or whether altered RBC parameters represent surrogate markers of concomitant conditions associated with risk of VTE, such as inflammation, is yet unknown [1].

Alterations of RDW are related to reduced RBC survival or disturbed erythropoiesis. It is known that inflammatory states and release of inflammatory cytokines, such as tumor necrosis factor (TNF)-alpha or interleukin-6, disrupt erythropoiesis [27], [28]. Therefore, RDW is closely related to a variety of conditions, such as anemia, inflammation, oxidative stress or malnutrition [12]. As these conditions are all frequently found in patients with cancer, we believe that a change in RDW is most probably a weaker risk factors for VTE in patients with cancer than other parameters that better reflect specific hypercoagulatory conditions. Cancer patients represent a group of patients with high VTE risk ranging up to 20% per year [29]. This high thrombotic risk is mediated and modulated by a multitude of factors, such as tumor biology [30], cancer spread [31], the presence of varicose veins [32] or different treatment modalities [33]. Tumor biology itself seems to have a major influence on risk of VTE, as cancer type, histological grading and stage of cancer disease have all been found to be risk factors for cancer-associated VTE [34]. Weak risk factors for VTE that are found in healthy individuals might therefore only have an only minor influence on the overall VTE risk in patients with cancer [35].

As a second finding of our study we confirmed the results of previous investigations showing that high RDW is associated with an increased risk of mortality [12]. For each 1% increase in RDW, the mortality risk increased by 11%. RDW was higher in patients with solid tumors who had metastases than in those with localized tumors, a finding that is consistent with a previous report on patients with lung cancer [6]. To account for this finding we adjusted for tumor stage in our multivariable model and still an independent association between high RDW and increased mortality was observed. Furthermore, consistent with the fact that anemia is a risk factor for poor prognosis in patients with cancer [36], in our study we found that increasing levels of erythrocyte count, hematocrit and hemoglobin were associated with an improved survival, and all to a similar extent. Underlying mechanisms that connect anemia and alterations of RBC parameters to increased mortality are not fully understood. Dysregulation of erythropoiesis due to oxidative stress and inflammatory states were proposed to play a role [12], and proinflammatory cytokines were shown to disrupt erythropoiesis. CRP is a known predictor of increased mortality [37], however, in the current study we found only a weak correlation between RDW and CRP, suggesting that inflammatory states, as measured by CRP levels, cannot sufficiently explain the association between RDW and increased mortality.

Some strengths and limitations of our study have to be addressed. First of all, levels of RDW are instrument-specific and can differ between laboratories and also populations. Therefore, the reference values used in our study cannot be applied to other populations and studies. However, we analyzed the association of RDW with risk of VTE and mortality using different cut-off values and RDW also as continuous variable. Furthermore, blood transfusions can affect RDW, but unfortunately in our study we do not have complete information about blood transfusions in study patients. However, the vast majority of study patients (73.5%) entered the study after newly diagnosis of a cancer disease and before beginning of treatment with chemotherapy, and therefore administration of blood transfusions prior to inclusion into our study that might affect RDW are very likely limited to a minority of study patients.

A further limitation of our study is the relatively small sample size and event rate within selected tumor types. We therefore cannot exclude a more pronounced effect of RDW on risk of VTE in these subgroups. Another limitation of our study is that we only recorded symptomatic VTE events, as we did not screen for VTE and we might therefore underestimate incidence of VTE in our study. A strength of our study is its prospective and observational design, which was chosen to specifically investigate risk factors for the development of VTE in patients with cancer.

In conclusion, the association between RDW and other RBC parameters and development of VTE in patients with cancer seems to be weak and not independent of other, already established risk factors, e.g. dissemination of cancer. We therefore suggest that RDW and other RBC parameters can rather not be considered as useful parameters for predicting risk of VTE in patients with cancer. As VTE is amongst the leading causes of death in patients with cancer [16], risk prediction of VTE might potentially be a major clinical benefit for cancer patients in terms of identifying patients at a very high risk, who might benefit from primary thromboprophylaxis. Therefore, the identification of parameters associated with high risk of VTE, especially of parameters that are readily available in routine praxis and inexpensive, such as parameters of the CBC, seems very reasonable. In a risk score published by Khorana et al. a low hemoglobin level was incorporated as a factor of increased VTE risk [15]. In contrast, our data suggest that several RBC parameters of the CBC, including hemoglobin levels, might not contribute to improved risk stratification of cancer-associated VTE, as they were not associated with VTE risk. Further studies are needed to clarify inconsistencies between studies. However, we could confirm findings of previous studies [12] showing that high RDW and low hemoglobin levels are significant and independent predictors of poor survival in patients with cancer and might therefore be relevant prognostic factors in these patients.

## Supporting Information

Table S1 **Characteristics of patients with RDW>16% and below.**
(PDF)
