# Supplementary material for: Red Cell Distribution Width and Other Red Blood Cell Parameters in Patients with Cancer: Association with Risk of Venous Thromboembolism and Mortality
Source: PLoS One. 2014 Oct 27;9(10):e111440. doi: 10.1371/journal.pone.0111440 (PMC4210186; doi:10.1371/journal.pone.0111440)
Supplement: Table S1 — Characteristics of patients with RDW>16% and below. (PDF) [file pone.0111440.s001.pdf]

Table S1. Characteristics of patients with RDW>16% and below. Continous variables are given by the mean and interquartile range. P-values for differences between groups are given.

	**RDW > 16% (n=188)**	**RDW ≤ 16% (n=1652)**	***p-value***
Hemoglobin, g/dl	10.5 (9.3-12.0)	13.2 (12.1-14.2)	*<0.001*
Erythrocytes, T/l	4.05 (3.40-4.45)	4.40 (4.10-4.70)	*<0.001*
Hematocrit, %	32.8 (29.8-36.8)	39.3 (36.3-41.7)	*<0.001*
MCV, fl	84.45 (77.80-90.65)	88.80 (85.90-91.80)	*<0.001*
MCH, pg	27.4 (24.6-29.5)	30.0 (28.9-31.1)	*<0.001*
MCHC, g/dl	32.1 (31.2-33.1)	33.7 (33.1-34.4)	*<0.001*
Leukocyte count, G/l	7.4 (5.7-10.9)	7.2 (5.7-9.5)	*0.307*
Platelet count, G/l	271 (177-364)	246 (198-306)	*0.191*
Use of ESA, n (%)	20 (10.6)	34 (2.1)	*<0.001*
Iron supplementary therapy, n (%)	12 (6.4)	12 (0.7)	*<0.001*
CRP, mg/dl	1.45 (0.25-5.07)	0.46 (0.15-1.40)	*<0.001*
